# Supplementary material for: Magnetic-Responsive Doxorubicin-Containing Materials Based on Fe3O4 Nanoparticles with a SiO2/PEG Shell and Study of Their Effects on Cancer Cell Lines
Source: Int J Mol Sci. 2022 Aug 13;23(16):9093. doi: 10.3390/ijms23169093 (PMC9409415; doi:10.3390/ijms23169093)
Supplement: Supplementary file 1 [file ijms-23-09093-s001.zip › ijms-1798158-supplementary.pdf]

Supplementary Materials

Magnetic-Responsive Doxorubicin-Containing Materials Based on Fe_3O_4 Nanoparticles with a SiO_2/PEG Shell and Study of Their Effects on Cancer Cell Lines

Alexander M. Demin [1,*], Alexander V. Vakhrushev [1], Alexandra G. Pershina [2,3], Marina S. Valova [1], Lina V. Efimova [2], Alexandra A. Syomchina [4], Mikhail A. Uimin [5], Artem Minin [5], Galina L. Levit [1], Victor P. Krasnov [1] and Valery N. Charushin [1]

[1] Postovsky Institute of Organic Synthesis, Russian Academy of Sciences (Ural Branch), Ekaterinburg 620108, Russia
[2] Siberian State Medical University, Tomsk 634050, Russia
[3] National Research Tomsk Polytechnic University, Tomsk 634050, Russia
[4] National Research Tomsk State University, Tomsk 634050, Russia
[5] Mikheev Institute of Metal Physics, Russian Academy of Sciences (Ural Branch), Ekaterinburg 620990, Russia

Calculation of the percentage of elements in nanocomposites based on EDX data

The EDX method does not give absolute values of the percentage (ω) of material elements, but only their percentage (ω') relative to all elements determined by this method. An EDX-7000 X-ray fluorescence spectrometer determines the percentage of elements starting from Na. Thus, the elements that are part of organic molecules (C, H, N) are not taken into account in the calculations of the device. Therefore, for the synthesized nanocomposites, we can only obtain ω' of Fe and Si. Nevertheless, by performing an elemental CHN analysis and finding ω of C, H, and N, we can take into account their content in the nanocomposite and, as a result, we can derive the formulas (S1)-(S2) to calculate ω of Fe and Si:

$$\omega_{Fe} = \frac{\omega'_{Fe} \times (100\% - \omega_C - \omega_H - \omega_N)}{(\omega'_{Fe} / \omega''_{Fe} + \omega'_{Si} / \omega''_{Si}) \times 100\%} \quad \text{(S1)}$$

$$\omega_{Si} = \frac{\omega'_{Si} \times (100\% - \omega_C - \omega_H - \omega_N)}{(\omega'_{Fe} / \omega''_{Fe} + \omega'_{Si} / \omega''_{Si}) \times 100\%} \quad \text{(S2)}$$

where ω is the wt. % of the corresponding element in the sample, which is found by the EA method; ω' is the wt. % of the corresponding element in the sample, which is determined by the EDX method; ω'' is the wt. % of Fe and Si in Fe_3O_4 or SiO_2, respectively ($(\omega''_{Fe_3O_4}$ = 72.37%, $(\omega''_{SiO_2}$ = 46.75%).

Calculation of the amount of SAPS in nanocomposites based on EA data

The amount of SAPS residues on the surface of nanocomposites was determined by formula (S3):

$$c = \frac{\omega_C}{\omega'' \times M} \quad \text{(S3)}$$

where c is the amount of SAPS residues on the surface of particles, mol per 1 g of MNPs; ω_C is the C wt. % in the samples of MNP **2–4**; ω''C is the C wt. % in the SAPS fragment (35.29%); *M* is the molar weight of SAPS fragment (238.37 g/mol).

Table S1. Elemental composition of the obtained MNPs@SiO_2 **1** and MNPs@TESPSA (MNPs **2–4**)

MNPs	EDX data		EA data	$Fe_3O_4 : SiO_2$ [a]	Amount of SAPS residues (mmol/g) [b]
	Fe (%)	Si (%)	C (%)		
**1**	77.12	22.88	0	69 : 31	0
**2**	76.16	23.84	1.74	67 : 33	0.21
**3**	75.77	24.23	0.90	67 : 33	0.11
**4**	75.77	24.23	0.75	67 : 33	0.09

[a] Calculated based on the EDX data using the equations (S1) and (S2); [b] calculated from the EA data using the equation (S3).

Table S2. Elemental composition of the obtained MNPs **6–8**

MNPs	EDX data		EA data	$Fe_3O_4 : SiO_2$ [a]
	Fe (%)	Si (%)	C (%)	
**6**	77.69	22.31	1.99	69 : 31
**7**	78.81	21.19	1.30	71 : 29
**8**	79.01	20.99	0.98	71 : 29

[a] Calculated based on the EDX data using the equations (S1) and (S2)

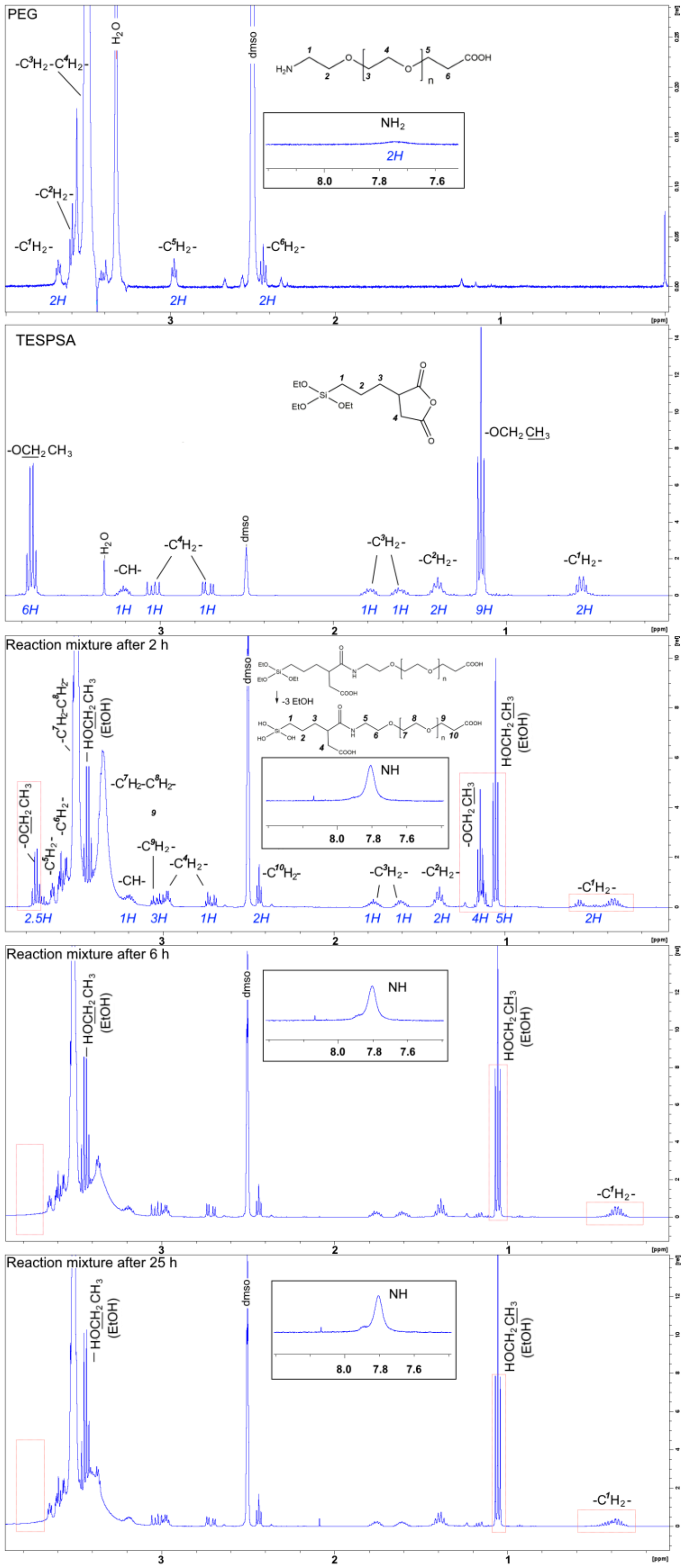

Figure S1. Selected fragments of ^{1}H NMR spectra (4.0–0.0 ppm) of the starting PEG and TESPSA, as well as the reaction mixture after their mixing in DMSO-d_6 in 2, 6, and 25 h (in insets, fragments of ^{1}H NMR spectra at $\delta = 8$ ppm). The red rectangles show the spectral regions corresponding to the signals characterizing the degree of hydrolysis of the TESPSA alkoxysilyl groups ($Si(OEt)_3$). The proton signals of the OEt group were significantly shifted as compared with the proton signals of EtOH formed as a result of hydrolysis. A shift of proton signals of CH_2-Si group was also observed before and after the hydrolysis of $Si(OEt)_3$.

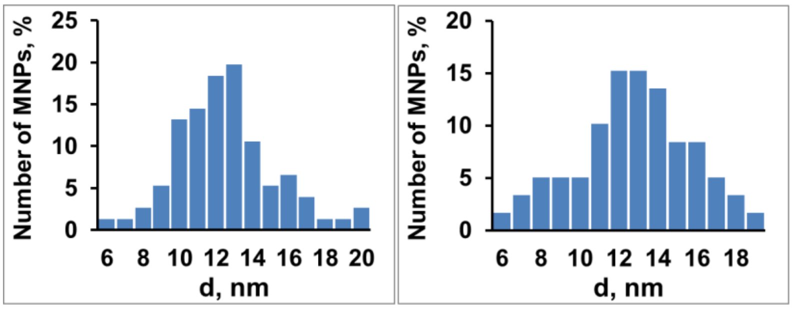

(a) (b)

Figure S2. The particle size distribution of the obtained MNPs (a) **6** and (b) **9** from TEM data.

Table S3. The electron diffraction data of the obtained MNPs **6** and **9**.

**Spot#**	**Crystallographic planes**	**d-Spacing, nm**		
		**Fe_3O_4***	**6**	**9**
1	220	0.2908	0.2908	0.2912
2	331	0.2523	0.2523	0.2523
3	400	0.2088	0.2088	0.2091
4	333	0.1607	0.1607	0.1612
5	440	0.1487	0.1487	0.1477

* JCPDS Card No. (79 - 0417) Magnetite – synthetic.

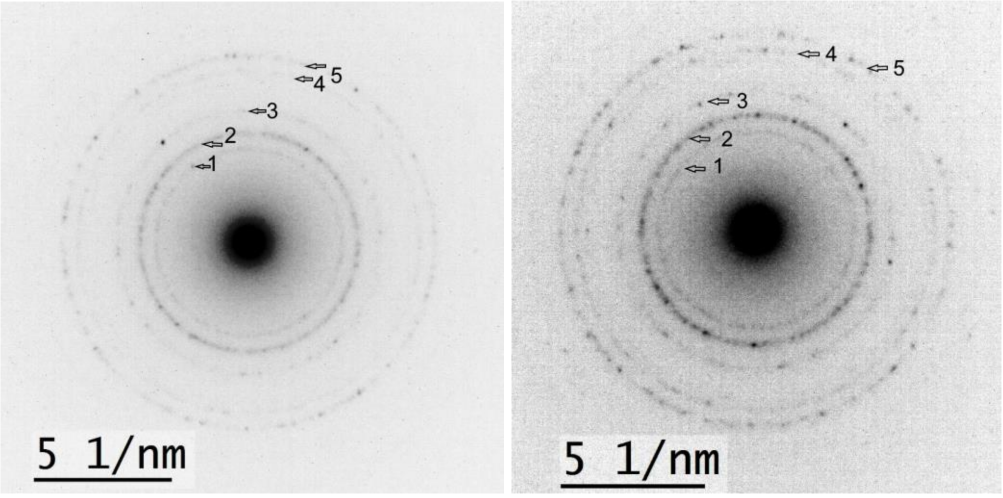

(a) (b)

Figure S3. Electron diffraction patterns of MNPs (a) **6** and (b) **9**.

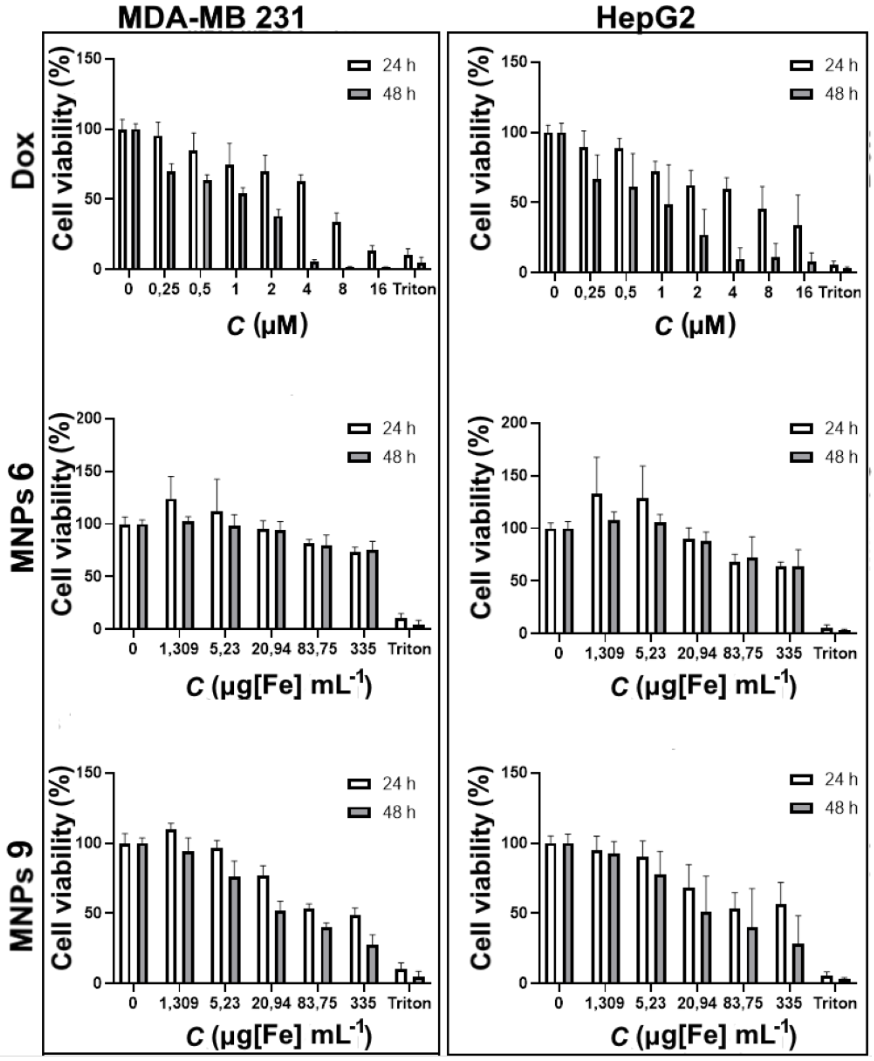

Figure S4. Dependence of the viability of MDA-MB231 and HepG2 cells on the concentration of Dox, MNPs **6** and MNPs **9** during incubation for 24 and 48 h (n = 3, N = 2).

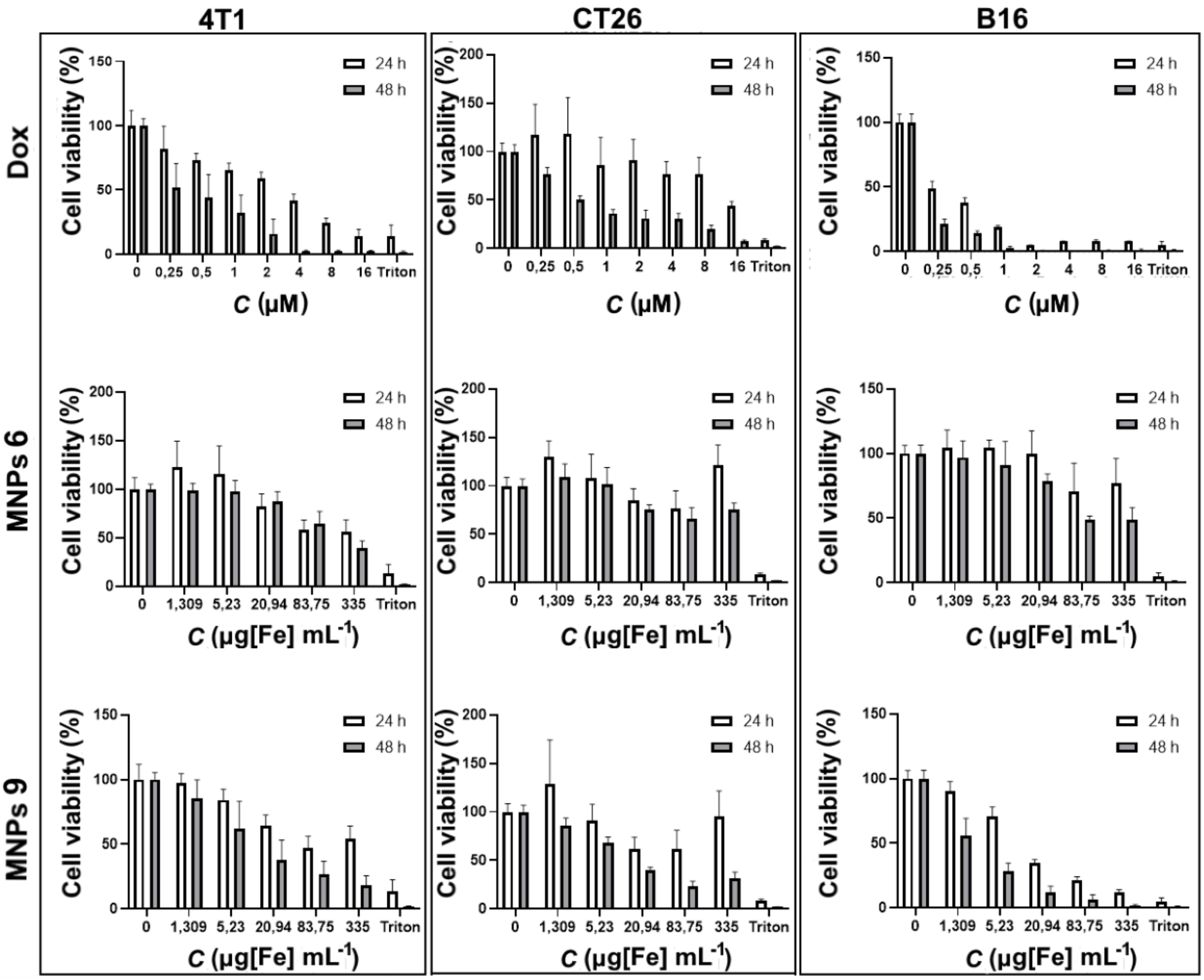

Figure S5. Dependence of the viability of 4T1, CT26 and B16 cells on the concentration of Dox, MNPs **6** and MNPs **9** during incubation for 24 and 48 h (n = 3, N = 2).
